# Mild Electrical Stimulation Increases Stress Resistance and Suppresses Fat Accumulation *via* Activation of LKB1-AMPK Signaling Pathway in *C. elegans*

**DOI:** 10.1371/journal.pone.0114690

**Published:** 2014-12-09

**Authors:** Shingo Matsuyama, Masataka Moriuchi, Mary Ann Suico, Shuichiro Yano, Saori Morino-Koga, Tsuyoshi Shuto, Kunitoshi Yamanaka, Tatsuya Kondo, Eiichi Araki, Hirofumi Kai

**Affiliations:** 1 Department of Molecular Medicine, Graduate School of Pharmaceutical Sciences, Kumamoto University, Kumamoto City, Japan; 2 Department of Molecular Cell Biology, Institute of Molecular Embryology and Genetics, Kumamoto University, Kumamoto City, Japan; 3 Department of Metabolic Medicine, Faculty of Life Sciences, Kumamoto University, Kumamoto City, Japan; Northwestern University Feinberg School of Medicine, United States of America

## Abstract

Electrical current at physiological strength has been applied as a therapeutic approach for various diseases. Several of our works showed that mild electrical stimulation (MES) at 0.1-ms pulse width has positive impact on organisms. But despite the growing evidence of the beneficial effects of MES, its effects on individual animals and the molecular underpinnings are poorly understood and rarely studied. Here, we examined the effects of MES on individual animal and its mechanisms by mainly using *Caenorhabditis elegans*, a powerful genetic model organism. Interestingly, MES increased stress resistance and suppressed excess fat accumulation in wild-type N2 worms but not in AMPK/AAK-2 and LKB1/PAR-4 mutant worms. MES promoted the nuclear localization of transcription factors DAF-16 and SKN-1 and consequently increased the expression of anti-stress genes, whereas MES inhibited the nuclear localization of SBP-1 and suppressed the expression of lipogenic genes. Moreover, we found that MES induced the activation of LKB1/PAR4-AMPK/AAK2 pathway in *C. elegans* and in several mammalian cell lines. The mitochondrial membrane potential and cellular ATP level were slightly and transiently decreased by MES leading to the activation of LKB1-AMPK signaling pathway. Together, we firstly and genetically demonstrated that MES exerts beneficial effects such as stress resistance and suppression of excess fat accumulation, via activation of LKB1-AMPK signaling pathway.

## Introduction

Endogenous and exogenous electrical current influence how proteins behave and interact with one another at cellular and organismal levels. Exogenous electrical stimulation has been studied using repetition frequency-, current wave form- or voltage-based condition [1]. Exogenous electrical stimulation has been used as therapeutic approach for the successful treatment of medical complaints such as inflammation, bone injury, nerve dysfunction and pain, and to improve abnormal blood circulation [2], [3]. Although the detailed cellular mechanisms for the positive effects of electrical stimulation remain elusive, it is thought that the beneficial effects can be attributed to the activation of signal transduction pathways [4].

We and others have reported that electrical stimulation activates PI(3)K-Akt signaling pathway resulting in wound healing and amelioration of hyperglycemia [5]–[7]. We have also reported that combination treatment of mild electrical stimulation (MES: pulse duration  = 0.1 millisecond, pulse number per second  = 55 pps) and heat shock (HS: 42°C), which can induce heat shock protein 72 (HSP72) expression, ameliorated hyperglycemia in diabetic mouse model, suppressed inflammatory cytokines in healthy male subjects, and decreased the proteinuria and renal inflammation in Alport syndrome mouse model [6], [8]–[11]. In these studies, MES affected the signaling pathways of PI(3)K-Akt, p38 mitogen-activated kinase (MAPK) and tumor suppressor p53 [10], [12], [13]. It is not surprising that, like other forms of physiological mechanical stresses such as shear stress, MES can activate the signal transduction pathways described above [4]. Furthermore, we recently reported that MES together with heat shock treatment reduced the visceral adiposity and inflammatory cytokines' expression, and improved glucose homeostasis in humans with metabolic syndrome or type 2 diabetes mellitus [14]. But despite the growing evidence of the beneficial effects of MES, the dissection of the mechanisms for MES effects is not fully understood. Especially, genetic knock-out model study is lacking.

In the present study, by mainly using *in vivo* genetic model system *C. elegans*, we genetically evaluated the effects of MES and its molecular mechanism. The distinctive advantage using *C. elegans* is that the experimental results in *C. elegans* can be predictive of outcomes in higher organisms due to its conserved biological processes [15]. Having previously established that MES exerted protective effects against some stresses and ameliorated metabolic dysfunction [16], we investigated here how MES affects stress resistance and fat metabolism, and which components regulate these phenomena. We hypothesized that MES can potentially impact on AMP-activated protein kinase (AMPK) signaling pathway, which plays a critical role for stress or damage responses and energy metabolism [17]. AMPK is activated by the upstream kinases LKB1 and CaMKKβ. A large variety of hormonal signals and cellular stress activates AMPK mostly through an increased cellular AMP: ATP ratios. In *C. elegans*, the α-subunit of AMPK, encoded by *aak-2*, also functions in stress responses, energy metabolism, dauer formation and life span [18], [19]. Metformin, one of the most known AMPK/AAK-2 activators, and overexpression of AAK-2 extended life span under oxidative stress [20]. However, it remains unclear whether MES activates AMPK.

Here, we showed that MES increased the median survival rate of wild-type *C. elegans* under heat and oxidative stress conditions, and suppressed excess fat accumulation when *C. elegans* were exposed to glucose-rich condition. However, the effects of MES were not observed in mutant strains of AMPK/AAK-2 and its upstream kinase LKB1/PAR-4. MES activated the AMPK signaling pathway via LKB1 but not CaMKKβ and this was likely due to a slight and transient inhibition of mitochondrial membrane potential and subsequent decline of cellular ATP levels. These findings firstly provide information on the molecular mechanisms of how MES induces stress resistance and suppresses excess fat accumulation at the organismal level.

## Materials & Methods

### Reagent and antibodies

STO-609, radicicol, rotenone and paraquat were from Sigma-Aldrich (St. Louis, MO). JC-1, Mitotracker Red CMXRos and CM-H_2_DCFDA were from Invitrogen (Carlsbard, CA). Antibodies against phospho-AMPK (Thr-172), AMPK, phospho-LKB1 (Ser-428) and LKB1 were from Cell Signaling Technology (Danvers, MA). β-actin antibody was from Santa Cruz Biotechnologies (Santa Cruz, CA). HRP-conjugated secondary antibodies were from Jackson Immuno Research Laboratories (West Grove, PA).

### Cell culture and in vitro MES treatment

Rat skeletal muscle cells (L6), human hepatocyte cells (HepG2) and human lung adenocarcinoma cells (A549) were maintained in Dulbecco's modified Eagle's medium (DMEM). Human cervical carcinoma cells (HeLa) were maintained in Minimum Essential medium (MEM). All cell lines were obtained from the American Type Cell Culture Collection (ATCC). The culture media contained 10% fetal bovine serum and antibiotics. Once L6 cells were grown to confluence, the medium was replaced with DMEM containing 2% fetal bovine serum to induce differentiation into myotubes. Cells were placed on 60-mm culture dishes and at 80% confluent were treated with MES as described previously [6]. Briefly, the culture plate with electrodes was carefully sealed and immersed in water bath at a temperature of 37°C. MES at 1 V/cm and 0.1 millisecond (ms) pulse duration was applied for 10 min. After MES treatment, the medium was immediately changed and the treated cells were incubated at 37°C until assay. For control of MES treatment, cells were sham treated by setting up the electrodes in similar manner as described above but without MES.

### Preparation of primary muscle cells and animal care

Satellite cells were obtained from lower hind limb muscle from 12-week old adult mouse. The muscles were excised from the lower limbs of mice, thoroughly minced, and digested in 0.2% type II collagenase (Wako, Japan) in Dulbecco's phosphate-buffered saline buffer with calcium (Invitrogen) for 30 min at 37°C. After digestion, the cells were collected via a 5-min centrifugation at 1800xg. The collected cells were exposed to trypsin-EDTA (0.25% with 1 mM EDTA) at 37°C for 10 min. The cells were diluted in 30 mL of culture medium containing 20% FBS, and 1% penicillin-streptomycin in DMEM, and filtered through 40-µm cell strainers (BD Biosciences, Franklin Lakes, NJ). Cells were grown in 5% CO_2_ at 37°C. Fibroblasts adhere to uncoated culture dishes more readily than muscle cells, so fibroblast contamination was minimized by pre-plating the cells onto 10-cm tissue culture dishes for 3 to 4 hr at 37°C. The unattached cells were centrifuged at 1800xg for 5 min, suspended in culture medium, plated onto dishes coated with 0.1% gelatin (Nacalai Tesque, Japan), and incubated at 37°C overnight. The attached cells were trypsinized, suspended in culture medium, and plated in culture dishes. After 1 to 2 days in culture, the culture medium was replaced with differentiation medium containing 2% FBS, 1% horse serum (Invitrogen), and antibiotics. Primary myotubes were used 4 to 5 days after induction of differentiation, when most cells were multinucleated. The mice used in this experiment were obtained from the Jackson Laboratories (Bar Harbor, Maine), and fed with food and water *ad libitum*. Mice were sacrificed by diethyl ether inhalation. The animal experiments were approved by the Animal Care and Use committee of Kumamoto University.

### Worm strains, growing conditions and in vivo MES treatment

All *C. elegans* strains used in this work were obtained from the Caenorhabditis Genetics Center (University of Minnesota), which is supported by the NIH NCRR. The following *C. elegans* strains used in the study are listed in Table 1. The *C. elegans* were maintained at 20°C on NGM plate (1.7% agar, 2.5 mg/mL peptone, 25 mM NaCl, 50 mM KH_2_PO_4_ pH 6.0, 5 µg/mL cholesterol, 1 mM CaCl_2_, 1 mM MgSO_4_) with fresh *Escherichia coli* OP50 as food source. Temperature-sensitive *par-4(it47)*, *par-4(it57)* mutants were maintained at 16°C and shifted to 25°C at the L1 stage when needed. Worm cultures were synchronized by the hypochlorite method and the resulting eggs were seeded onto NGM agar plates. For MES treatment, worms were collected from the plates with M9 buffer (42 mM Na_2_HPO_4_, 22 mM KH_2_PO_4_, 86 mM NaCl, 1 mM MgSO_4_ •7H_2_O), transferred to 60-mm dishes. The dishes containing worm suspension were carefully covered with lid with electrodes and kept at a temperature of 20°C. MES at 2 V/cm and 0.1 millisecond (ms) pulse duration was applied for 20 min. After MES treatment, worm suspensions were immediately collected to tubes, centrifuged and transferred to NGM plate, and then incubated at proper temperature for each strain until assay. For control of MES treatment, worm suspensions were sham treated by setting up the electrodes in a similar manner as described above but without MES.

**Table 1:** List of C. elegans used in this study.

Strain	Genotype
N2	*wild type*
TG38	*aak-2(gt33)*
RB754	*aak-2(ok524)*
KK184	*par-4(it47)*
KK300	*par-4(it57)*
CF1139	*daf-16(mu86) I; muIs61 [(pKL78) daf16::GFP + rol-6(su1006)]*
LD1	*idIs7 [skn-1B/C::GFP + pRF4(rol-6(su1006))]*
OG497	*drSi13 [hsf-1p::hsf-1::GFP::unc-54 3'UTR + Cbr-unc-119(+)] II*
CE548	*sbp-1(ep79) III; epEx141 [sbp-1::GFP::SBP-1 + rol-6(su1006)]*
CF1553	*muIs84 [(pAD76) sod-3p::GFP + rol-6]*
CL2070	*dvIs70 [hsp-16.2p::GFP + rol-6(su1006)]*

### Heat stress resistance assays

Worms were treated once a day with MES (20 min) for 3 days at larval stage. After the last treatment, worms were incubated under heat (30°C) stress condition. Worms were then scored as dead or alive by tapping them with a platinum wire every 24 hr. Worms that died with larvae inside (eggs hatched before being laid) or those in which the intestine extruded from the vulva were removed from the sample. Each time point is the average of three independent experiments with 80–100 animals for each strain.

### Oxidative stress resistance assays

Worms were treated once a day with MES (20 min) for 3 days at larval stage. After the last treatment, worms were transferred onto plates that included 4 mM paraquat (Sigma-Aldrich) in NGM agar plates. N2, *aak-2(gt33)* and *aak-2(ok524)* mutants were incubated on these plates at 20°C. Temperature-sensitive *par-4(it47)*, *par-4(it57)* mutants were incubated on these plates at 25°C. Worms were then scored as dead or alive by tapping them with a platinum wire every 24 hr. Worms that died with larvae inside (eggs hatched before being laid) or those in which the intestine extruded from the vulva were removed from the sample. Each time point is the average of three independent experiments with 80–100 animals for each strain.

### Body bending assay

The number of body bends was counted during (time point  = 0, 5, 15, 20 min) and after MES treatment for 20 seconds in M9 buffer. The movies were recorded using BioRevo BZ-9000 (Keyence, Japan). A body bend was defined as change in direction of the bend at the mid-body of a worm.

### Pharyngeal pumping assay

The average pharyngeal pumping rates of worms while feeding on culture plates were recorded using BioRevo BZ-9000 (Keyence) per 5 seconds. Pharyngeal grinder movements in any axis were scored as a pumping event.

### Western blotting analysis

Worms were collected from the plates with M9 buffer, transferred to tubes, centrifuged and washed three times to eliminate bacteria. The final worm pellet was resuspended in lysis buffer (25 mM HEPES, 10 mM Na_4_P_2_O_7_ 10H_2_O, 100 mM NaF, 5 mM EDTA, 2 mM Na_3_VO_4_, 1% Triton X-100) containing 1% protease inhibitor (PI) cocktail (Sigma-Aldrich). The samples were sonicated 3 times on ice for 10 seconds and centrifuged for 15 minutes at 12,000 rpm and 4°C to eliminate the cuticles. Cells were washed with PBS and lysed in lysis buffer. Worms and cell protein lysates were subjected to SDS-PAGE on 10% polyacrylamide gel. Proteins were electroblotted to PVDF membrane (Millipore Corp, Bedford, MA). Blots were reacted with specific primary antibodies and with respective HRP-conjugated secondary antibodies obtained from the Jackson ImmunoResearch Laboratories. SuperSignal WestPico chemiluminescence substrate (Thermo Scientific Inc., Waltham, MA) and ECL chemiluminescence reagent were used (Amersham Pharmacia Biotech, UK) for visualizing the blots.

### Real-time Quantitative RT-PCR analysis (Q-PCR)

Worms were collected from the plates with M9 buffer, transferred to tubes, centrifuged and washed three times to eliminate bacteria. The final worm pellet was suspended with RNAiso reagent (Takara Bio Inc., Japan), and total RNA was isolated following the manufacturer's instructions. Real-time quantitative reverse transcriptase polymerase chain reaction (RT-PCR) analyses for the indicated genes and internal control β-actin were carried out with SYBR Green Master Mix (Applied Biosystems, Foster City, CA) following the manufacturer's instructions. The Ct values for each gene amplification were normalized by subtracting the Ct value calculated for β-actin. The oligonucleotide primers used in the real-time quantitative PCR are listed in Table 2.

**Table 2:** List of primers used for qPCR.

	Primer sequence	
Gene	Sense	Anti-sense
C. elegans		
*sod-1*	5′-ttctcactcaggtctccaacgcgat-3′	5′-ctggtcattttcggacttctgtgtg-3′
*sod-2*	5′-ttcacgaggcggtctccaaaggaaa-3′	5′-tggttctcctccgtcctttgccaaa-3′
*sod-3*	5′-ttgttcaaccggttgcgggagttct-3′	5′-agaactcccgcaaccggttgaacaa-3′
*sod-4*	5′-gaagcttaacggatcggtttccgga-3′	5′-tgcaccatggctcagcttatgagga-3′
*sod-5*	5′-ttggcttacccagaaagccgaaggt-3′	5′-gacgtacatccatcggttgagtctc-3′
*hsp-70*	5′-gaaaggttgaaatcctcgcgaactc-3′	5′-tccggattacgagcggcttgatctt-3′
*hsp-12.6*	5′-tggagttgtcaatgtcctcg-3′	5′-gacttcaatctcttttgggagg-3′
*hsp-16.1*	5′-tgcagaggctctccatctgaatct-3′	5′-tcttctggcttgaactgcgagaca-3′
*hsp-16.2*	5′-ctccagtctgcagaatctctccat-3′	5′-gtgagacgttgagattgatggca-3′
*sbp-1*	5′-cagcgaaattcggaacctggatctc-3′	5′-tcggcggacagttcctgatcagtga-3′
*nhr-49*	5′-gatcgcaaatctttgcaaacggcag-3′	5′-tcagatctacctcggttgtgcatgg-3′
*fat-7*	5′-ccagagaaagcactatttcccactg-3′	5′-tgttgttgcaccaagtggcgtgaag-3′
*acs-2*	5′-taccgagacatcccctctagtcacc-3′	5′-gctctcctttagctccagttggcac-3′
*gei-7*	5′-ggaaatcctttcgctcaccgcccaa-3′	5′-atatcagcctgaacttggttgcgct-3′
*β-actin*	5′-ccaagagaggtatccttaccctcaa-3′	5′-tcttctggggcaacacgaagctcat-3′

### Making of *C. elegans* obesity model

Worms were cultured in 10 mM D-glucose-containing NGM plates for 3 days at larval stage. Then the worms were subjected to Nile red analysis for determining fat accumulation.

### Worm fixation and Nile red staining

Worms were washed twice with PBS and suspended in PBS buffer to which an equal volume of 2× MRWB buffer (160 mM KCl, 40 mM NaCl, 14 mM Na_2_EGTA, 1 mM spermidine, 30 mM PIPES pH 7.4, 50% methanol, 2% paraformaldehyde) was added. The worms were taken through 3 freeze-thaw cycles between dry ice/ethanol and warm (37°C) water, followed by washing in PBS to remove paraformaldehyde. Nile red stock solution (500 µg/mL) was made by dissolving in acetone. It was then diluted in water (100 ng/mL final concentration) and worms were incubated overnight in the working solution on a shaker at room temperature. Stained worms were washed three times with PBS and mounted on a thick layer of half-dried 4% agarose pad on microscopic glass slides, covered with cover slip. Pictures were taken using BioRevo BZ-9000 (Keyence). ImageJ software (NIH; http://rsbweb.nih.gov/ij/) was used to quantify image fluorescence obtained using the microscope. Exposure times were kept constant within each trial.

### GFP fluorescence imaging and quantification

Worms were washed twice with M9 buffer and fixed with 25 mM sodium azide. Worms were mounted on a thick layer of half-dried 4% agarose pad on microscopic glass slides, covered with cover slip. Pictures were taken using BioRevo BZ-9000 (Keyence). ImageJ software was used to quantify image fluorescence obtained using the microscope. Exposure times were kept constant within each trial.

### ATP content measurement

ATP contents were measured by using the ATP bioluminescence assay kit HSII (Roche Applied Science, Germany) according to the manufacturer's instructions. Briefly, cell and worms were washed with PBS and lysed with lysis buffer. The lysates were boiled for 15 min and centrifuged at 15,000 g for 5 min. The supernatants were recovered as samples. Fifty microliters of each sample or standard was transferred into a disposable cuvette and 50 µL of luciferase reagent was added to it. After mixing, the light emitted was measured by using a TD−20/20 Turner Designs luminometer (Promega, Madison, WI). The blank value (no ATP) was subtracted from each sample's raw data. Final ATP concentrations were calculated from the linear part of a standard curve and normalized with each sample's protein concentration measured by BCA method.

### JC-1 mitochondrial membrane potential assay

Mitochondrial membrane potential (Δψ_m_) was assessed by using JC-1, a cationic dye that exhibits potential-dependent accumulation and formation of red dye fluorescent J-aggregates in mitochondria, which is indicated by a fluorescence emission shift from green (525 nm) to red (590 nm). After MES treatment, cells were washed with PBS, trypsinized, and resuspended in culture medium. Each sample was loaded with 10 µM JC-1 for 30 min at 37°C. After being loaded, cells were washed and resuspended in PBS. Fluorescence was determined by flow cytometry (FACSCalibur, BD Biosciences). The JC-1 monomer (green) and the J-aggregates (red) were detected separately in FL1 (emission, 525 nm) and FL2 (emission, 590 nm) channels, respectively. Δψ_m_ is presented as red to green fluorescence intensity ratio. Data from the experiments were analyzed by CellQuest software (BD Biosciences).

### Mitotracker staining

Mitotracker Red CMXRos (Invitrogen) dye was diluted in DMSO at 1 mM concentration and frozen at −20°C. Before staining, stocks were diluted in M9 buffer at 10 µM. Worms were washed twice with M9 buffer and transferred into staining solution and stained for 20 min. After staining, worms were washed twice and mounted on a thick layer of half-dried 4% agarose microscopic pad on glass slides. Pictures were taken using BioRevo BZ-9000 (Keyence). ImageJ software was used to quantify image fluorescence obtained using the microscope. Exposure times were kept constant within each trial.

### LDH assay

Cells were assayed for LDH release according to the protocol as described previously [21]. After cells were treated with MES, culture media were isolated and centrifuged at 12,000 rpm for 15 min. Supernatants were recovered, and cell pellets were lysed by adding 1% Triton X-100 solutions for 30 min at 37°C. The remaining attached cells in plates were also lysed with 1% Triton X-100 solution. Culture media, cell pellets and lysates were subjected to LDH assay using the Cytotoxicity Detection Kit (Roche Applied Science, Germany) according to the manufacturer's instructions. LDH release was expressed as percentage of the LDH in the medium and pellet over the total LDH (medium, pellet and lysate).

### ROS detection

ROS production was detected using 2′,7′-dichlorodihydro-fluorescein diacetate acetyl ester (CM-H_2_DCFDA) (Invitrogen) dye according to the manufacturer's instructions. Pictures were taken using BioRevo BZ-9000 (Keyence). ImageJ software was used to quantify image fluorescence obtained using the microscope. Exposure times were kept constant within each trial.

## Results

### MES increased stress resistance in wild type N2 *C. elegans*

**Figure 1:**
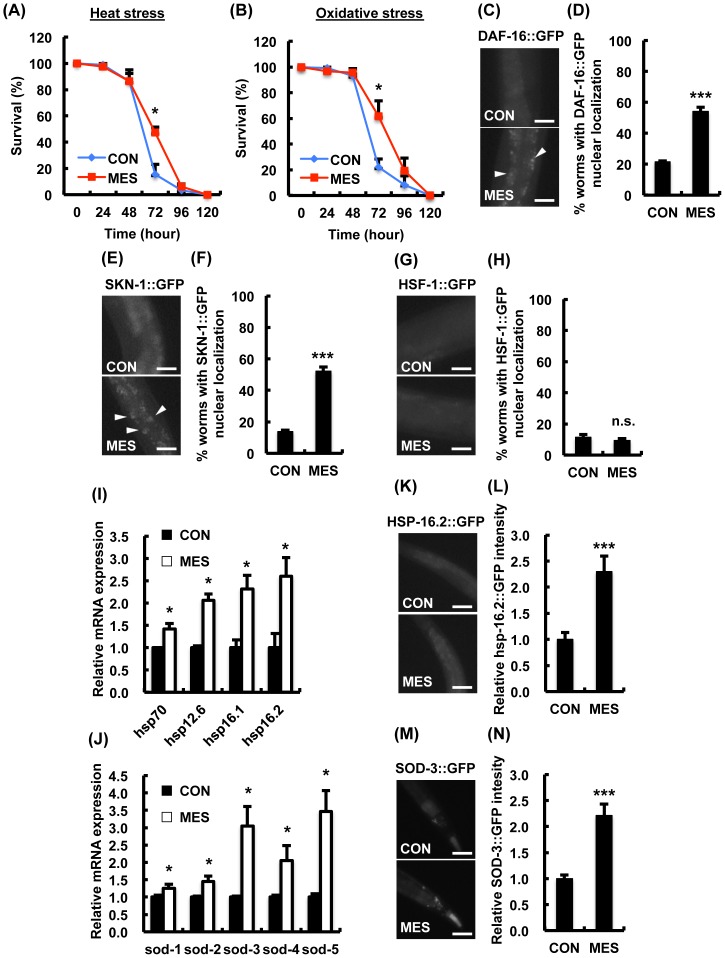
MES increases stress resistance in wild type N2 worms. (A, B) N2 worms were treated once a day with MES (0.1 ms. 2 V/cm, 55 pps) for 20 min at larval stage. After the last treatment, worms were incubated under (A) heat stress (30°C) or (B) oxidative stress (4 mM paraquat) condition. Worms' survival was checked every 24 hr. Each time point is the average of three independent experiments using 80–100 animals per group for each experiment. Data are presented as mean ± SE. ^*^P<0.05, assessed by unpaired Student t-test. (C-H) Worms expressing GFP reporter were treated once a day with MES for 20 min at larval stage. After the last treatment, images of GFP (DAF-16::GFP, SKN-1::GFP and HSF-1::GFP) were obtained using a fluorescent microscope. Percentage of worms with GFP nuclear localization (white arrowhead) was quantified. Data are presented as mean ± SE. ^***^P<0.001 versus control, assessed by unpaired t-test. Scale bars, 25 µm. (I, J) Wild type N2 worms were treated once a day with MES for 20 min at larval stage. After the last treatment, total RNA was extracted and subjected to quantitative real-time PCR. Data are representative of two independent experiments using approximately 200 animals per group. Data are presented as mean ± SE. ^*^P<0.05, assessed by unpaired Student t-test. (K-N) Worms expressing GFP reporter were treated once a day with MES for 20 min at larval stage. After the last treatment, images of GFP (HSP-16.2, SOD-3::GFP) were obtained using a fluorescent microscope. Relative fluorescent intensity of GFP was quantified. Data are presented as mean ± SE. ^***^P<0.001, assessed by unpaired Student t-test. Scale bars, 25 µm. For GFP fluorescence experiments (C–H and K–N), data shown are representative of two independent experiments using 40–50 animals per group.

To determine whether MES has protective effect against stress, we first monitored the survival rates of MES-treated wild type N2 worms exposed to lethal stress. We used MES conditions, which we previously demonstrated to have impact in cells and mice [6], [10], [13]. Compared with control, MES increased the survival rate of worms at 72 hr by approximately 30% under heat stress and 40% under oxidative stress (Fig. 1A–B). These effects were pronounced in mid life without maximum survival rate extension, indicating that MES specifically impacts median survival rate under stress conditions.

We next evaluated whether MES activates heat and oxidative stress-responsive transcription factors. Specifically, *C. elegans* FOXO class forkhead transcription factor DAF-16, SKN-1 and heat shock factor-1 (HSF-1) are known to increase stress resistance and extend life span [19]. In response to stress, these transcription factors translocate into the nucleus and activate target gene expression. We utilized *C. elegans* expressing GFP translational reporter, namely, DAF-16::GFP, SKN-1::GFP and HSF-1::GFP. The localization of these transcription factors after MES treatment was assessed by fluorescent microscopy, and the number of worms with GFP fluorescence in the nuclei was counted. MES increased the nuclear expression of DAF-16::GFP (Fig. 1C–D) and SKN-1::GFP (Fig. 1E–F), but not HSF-1::GFP (Fig. 1G–H).

We also examined the induction of anti-heat stress gene *hsp*s and anti-oxidative stress gene *sod*s, which are target genes of DAF-16 and SKN-1 [22]. MES-treated wild-type N2 worms showed higher expression of *hsp* and *sod* genes than non-treated worms (Fig. 1I–J). Furthermore, the protein expression was increased in MES-treated worms, assessed using GFP fluorescence of HSP-16.2 and SOD-3 (Fig. 1K–N). These results suggested that MES-induced stress resistance involves the activation of DAF-16, SKN-1 and their target genes.

### MES suppressed excess fat accumulation in wild type N2 *C. elegans* under high glucose condition

**Figure 2:**
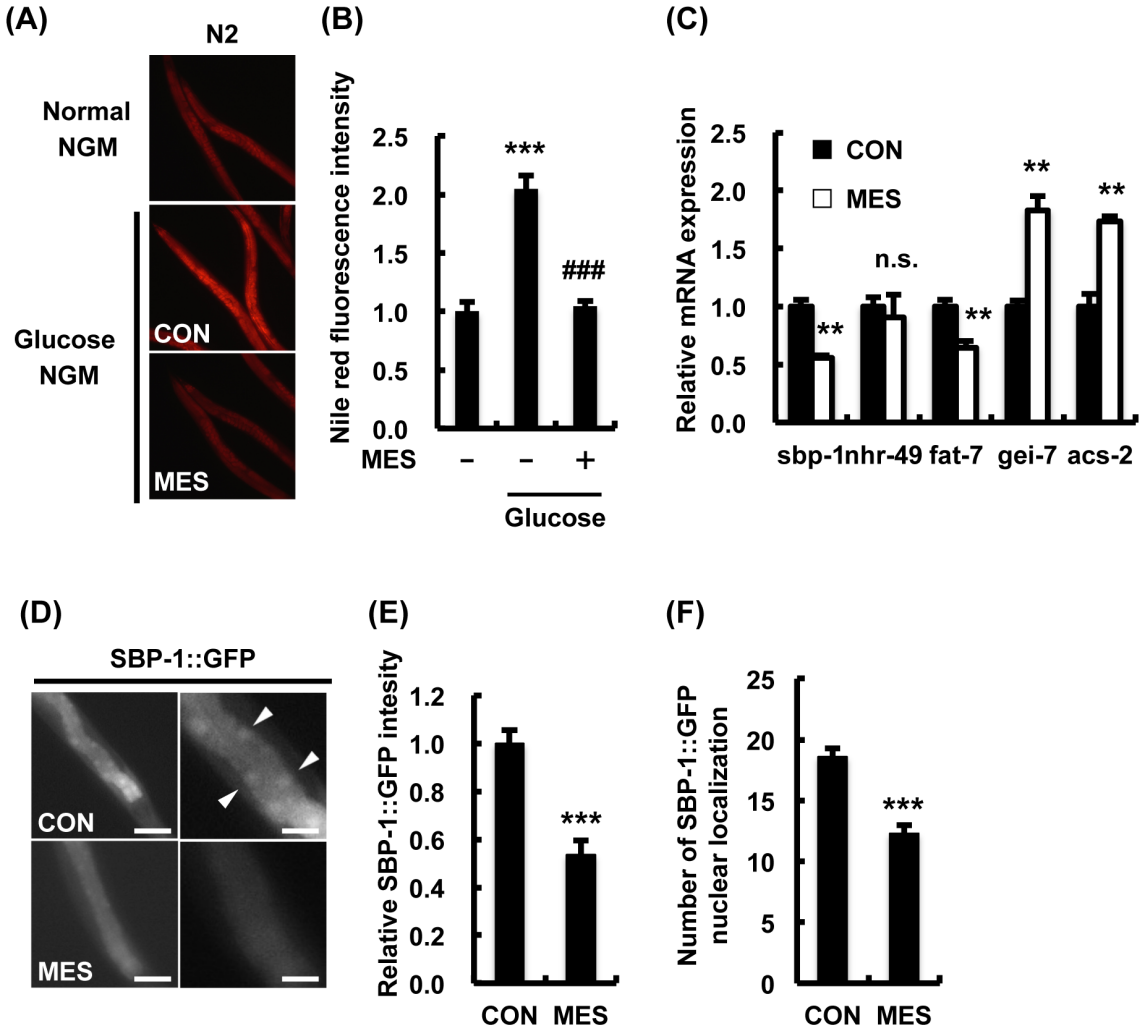
MES suppresses excess fat accumulation in wild type N2 worms. (A) N2 worms were bred on NGM plates with or without D-glucose (10 mM) and treated once a day with MES (0.1 ms, 2 V/cm, 55 pps) for 20 min at larval stage. After the last treatment, worms were stained with Nile red and observed under a fluorescent microscope. (B) Relative fluorescent intensity of Nile red in (A) was quantified. Data are presented as mean ± SE. ^***^P<0.001 versus control without D-glucose, ^###^P<0.0001 versus control with D-glucose, assessed by one-way ANOVA. (C) Wild type N2 worms were bred on NGM plates with glucose (10 mM) and treated once a day with MES for 20 min at larval stage. After the last treatment, total RNA was extracted and subjected to quantitative real-time PCR. The mRNA levels were normalized to the level of β-actin (internal control). Data are representative of two independent experiments using approximately 200 animals per group. Data are presented as mean ± SE. ^**^P<0.01 versus control, assessed by unpaired t-test. n.s., not significant. (D) Worms expressing SBP-1::GFP reporter were treated once a day with MES for 20 min at larval stage. After the last treatment, images of GFP were obtained using a fluorescent microscope. Scale bar in left panels, 100 µm; right panels, 25 µm (E) Percentage of worms with GFP nuclear localization (white arrowhead in (D)) and (F) number of GFP nuclear foci were quantified. Data are presented as mean ± SE. ^***^P<0.001 versus control, assessed by unpaired t-test. For fluorescence experiments (A–B and D–F), data shown are representative of two independent experiments using 40–50 animals per group.

Because our previous studies demonstrated that applied electrical stimulation ameliorated metabolic dysfunction in mammals [23], we examined the effects of MES on excess fat accumulation. First, we produced *C. elegans* obesity model as previously reported [24]. Nile red staining analysis indicated a considerable increase of fat accumulation in N2 worms in glucose-rich condition. Interestingly, MES suppressed excess fat accumulation in worms (Fig. 2A–B). MES also suppressed the expression of fatty acid synthesis-related genes *sbp-1* and *fat-7* and increased the expression of β-oxidation-related genes *gei-7* and *acs-2* (Fig. 2C). SBP-1, a homolog of the mammalian transcription factor SREBP-1c, is a key factor associated with fat accumulation and cholesterol synthesis. Here, we utilized the worm expressing SBP-1::GFP translational reporter. MES-treated worms showed lower fluorescence intensity and less nuclear localization of SBP-1::GFP compared with control worms, indicating decreased protein expression and transcriptional activity of SBP-1 (Fig. 2D–F). These results suggest that MES suppressed excess fat accumulation by decreasing SBP-1 expression and transcriptional activity, subsequently altering the expression of fatty acid metabolism-related genes.

### MES did not increase stress resistance or suppress excess fat accumulation in AAK-2/AMPK mutant worms

**Figure 3:**
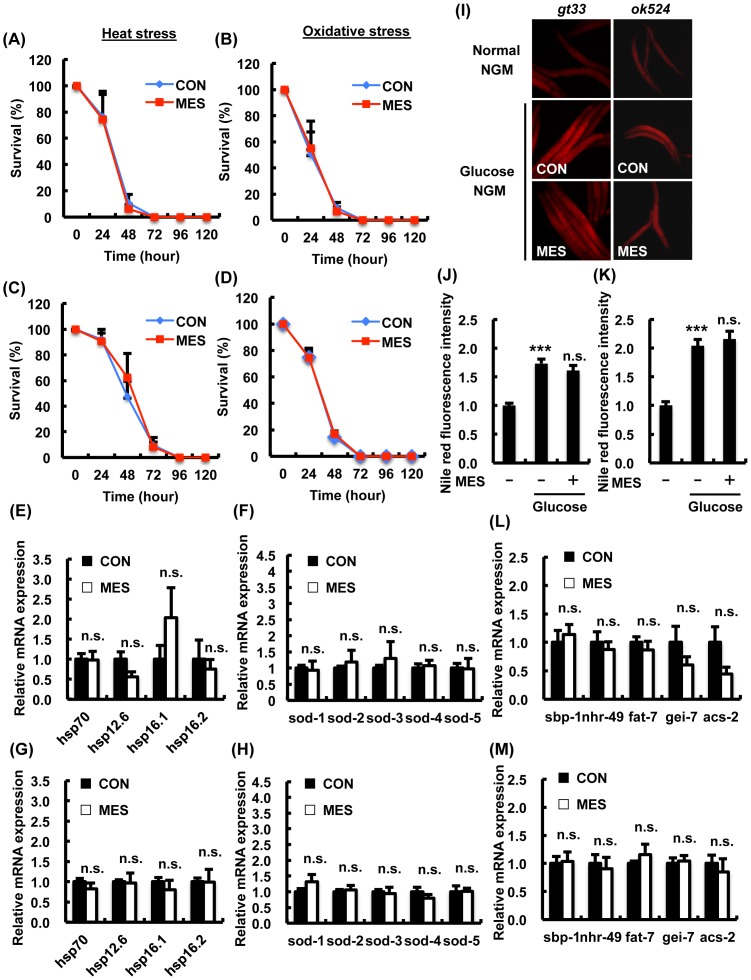
MES-induced phenotypes are not observed in AMPK homolog *aak-2* mutant worms. (A–D) *aak-2* mutant worms (A, B: *gt33*; C, D: *ok524)* were treated once a day with MES (0.1 ms, 2 V/cm, 55 pps) for 20 min at larval stage. After the last treatment, worms were incubated under (A, C) heat stress (30°C) or (B, D) oxidative stress (4 mM paraquat) condition. Worms' survival was checked every 24 hr. Each time point is the average of three independent experiments using 80–100 animals per group. (E-H) *aak-2* mutant worms (E, F: *gt33*; G, H: *ok524*) worms were bred on NGM plates and treated once a day with MES for 20 min at larval stage. After the last treatment, total RNA was extracted and subjected to quantitative real-time PCR. The mRNA levels were normalized to the level of β-actin (internal control). Data are presented as mean ± SE. n.s. versus control, assessed by unpaired Student t-test. (I) *aak-2(gt33, ok524)* worms were bred on NGM plates and treated once a day with MES for 20 min at larval stage. After the last treatment, worms were stained with Nile red and observed using a fluorescent microscope. (J, K) Relative fluorescence intensity of Nile red for (J) *gt33* and (K) *ok524* was quantified. Data are representative of two independent experiments using 40–50 animals per group. Data are presented as mean ± SE. ^***^P<0.001 versus control without D-glucose, n.s. versus control with D-glucose, assessed by one-way ANOVA. (L, M) *aak-2* mutant worms (L: *gt33*; M: *ok524*) were bred on NGM plates containing D-glucose (10 mM) and treated once a day with MES for 20 min at larval stage. After the last treatment, total RNA was extracted and subjected to quantitative real-time PCR. The mRNA levels were normalized to the level of β-actin (internal control). Data are presented as mean ± SE. n.s. versus control, assessed by unpaired t-test. n.s., not significant. For mRNA analysis (E–H and L–M), data shown are representative of two independent experiments using approximately 200 animals per group.

We next identified the key molecules that mediate the physiological effects of MES on *C. elegans*. The *C. elegans aak-2*, which encodes the homolog of the catalytic α-subunit of mammalian AMPK, acts as a key gene that regulates stress resistance, energy metabolism, dauer formation and life span [18]. To investigate the involvement of AAK-2, we utilized two *aak-2* loss-of-function mutants. The deleted regions of *aak-2(gt33)* and *aak-2(ok524)* contain a phosphorylation site at Thr-243 corresponding to Thr-172 of human AMPKα subunit, which is embedded in conserved sequences [25]. We monitored the survival rates of MES-treated *aak-2* mutant worms under stress conditions. As previously reported, *aak-2* mutant worms showed hypersensitivity to heat or oxidative stress (Fig. 3A-D) [26]. MES did not extend the survival rate of *aak-2(gt33)* (Fig. 3A-B) and *aak-2(ok524)* (Fig. 3C-D) mutant worms under heat stress or oxidative stress conditions. The expressions of anti-heat stress genes *hsp*s and anti-oxidative stress genes *sod*s in *aak-2(gt33)* (Fig. 3E-F) and *aak-2(ok524)* (Fig. 3G-H) mutant worms were not induced by MES. We also examined the effects of MES on excess fat accumulation in *aak-2* mutants. The level of fat accumulation in MES-treated *aak-2* mutant worms was relatively similar to non-treated control in high glucose condition (Fig. 3I–K). The expression of fatty acid synthesis-related genes such as *sbp-1* and β-oxidation-related genes remained unchanged in MES-treated *aak-2* mutant worms compared with control (Fig. 3L–M). These results indicated that *aak-2* plays an important role in MES-induced stress resistance and suppression of excess fat accumulation.

### MES did not increase stress resistance or suppress excess fat accumulation in AMPK kinase LKB1/PAR-4 mutant worms

**Figure 4:**
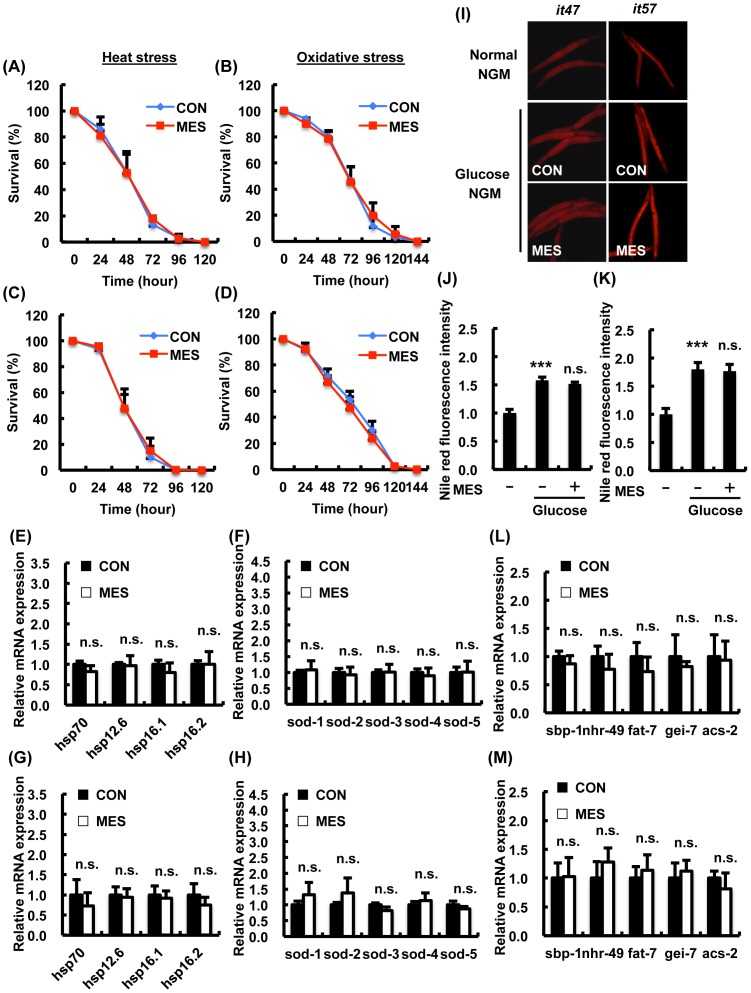
MES-induced phenotypes are not observed in AMPK-kinase LKB1 homolog *par-4* mutant worms. (A–D) *par-4* mutant worms (A, B: *it47*; C, D: *it57)* were treated once a day with MES (0.1 ms, 2 V/cm, 55 pps) for 20 min at larval stage. After the last treatment, worms were incubated under (A, C) heat stress (30°C) or (B, D) oxidative stress (4 mM paraquat) condition. Worms survival were checked every 24 hr. Each time point is the average of three independent experiments using 80–100 animals per group. (E–H) *par-4 mutant* worms (E, F: *it47*; G, H: *it57*) were bred on NGM plates and treated once a day with MES for 20 min at larval stage. After the last treatment, total RNA was extracted and subjected to quantitative real-time PCR. The mRNA levels were normalized to the level of β-actin (internal control). Data are presented as mean ± SE. n.s. versus control, assessed by unpaired t-test. (I) *par-4(it47, it57)* worms were bred on NGM plates containing D-glucose (10 mM) and treated once a day with MES for 20 min at larval stage. After the last treatment, worms were stained with Nile red and observed using a fluorescent microscope. (J, K) Relative fluorescence intensity of Nile red for (J) *it47* and (K) *it57* was quantified. Data are representative of two independent experiments using 40–50 animals per group. Data are presented as mean ± SE. ^***^P<0.001 versus control without D-glucose, n.s. versus control with D-glucose, assessed by one-way ANOVA. (L, M) *par-4* mutant worms (L*: it47*; M: *it57*) were bred on NGM plates containing D-glucose (10 mM) and treated once a day with MES for 20 min at larval stage. After the last treatment, total RNA was extracted and subjected to quantitative real-time PCR. The mRNA levels were normalized to the level of β-actin (internal control). Data are presented as mean ± SE. n.s. versus control, assessed by unpaired t-test. n.s., not significant. For mRNA analysis (E–H and L–M), data shown are representative of two independent experiments using approximately 200 animals per group.

It was reported that AAK-2 was activated by the *C. elegans* LKB1 homolog PAR-4 to exhibit stress resistance, energy metabolism and normal behavior [26]. Thus, we investigated whether PAR-4 is important for the effects of MES using two *par-4* mutant alleles *par-4(it47)* and *par-4(it57)*. *par-4* mutant worms exhibited hypersensitivity to heat or oxidative stress (Fig. 4A–D) similarly to *aak-2* mutant worms as previously shown [27]. MES did not extend the survival rate of *par-4(it47)* (Fig. 4A–B) and *par-4(it57)* (Fig. 4C-D) mutant worms under heat stress or oxidative stress conditions. MES did not induce the expression of anti-heat stress genes *hsp*s and anti-oxidative stress genes *sod*s in *par-4(it47)* (Fig. 4E–F) and *par-4(it57)* (Fig. 4G–H) mutant worms. MES did not suppress excess fat accumulation in *par-4* mutant worms in high glucose condition (Fig. 4I–K). MES did not change the expression of fatty acid synthesis-related genes and β-oxidation-related genes in *par-4* mutant worms compared with control (Fig. 4L–M). These results suggest that PAR-4/LKB1, the upstream kinase of AAK-2/AMPK, is required for MES-induced effects.

### LKB1-AMPK pathway is activated by MES in *C. elegans* and mammalian cells

**Figure 5:**
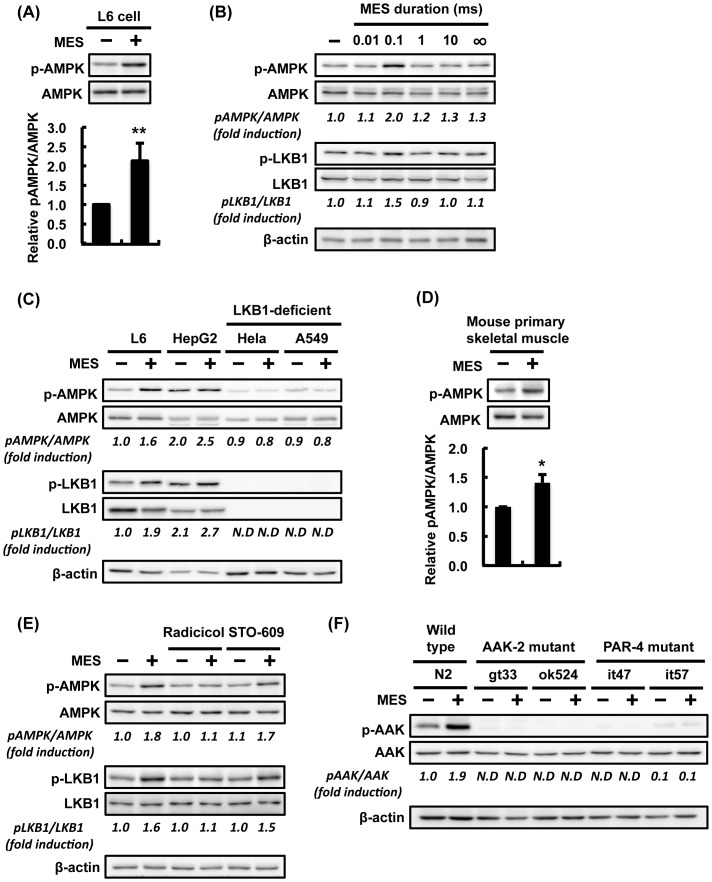
MES induces AMPK activation via LKB1-dependent pathway. (A) Differentiated L6 cells were treated with MES (0.1 ms, 1 V/cm, 55 pps) for 10 min. Cell lysates were extracted 2 hr after MES treatment. Phospho-AMPK (Thr-172) and AMPK were detected by Western Blotting analysis. Relative amount of p-AMPK was normalized to total AMPK. Data are presented as mean ± SE (n = 3). ^**^P<0.01 versus control, assessed by unpaired Student t-test. (B) Differentiated L6 cells were treated with MES (1 V/cm, 55 pps) at various pulse widths (0.01, 0.1, 1 and 10 ms) or in the absence of pulse (∞ ms) for 10 min. Cell lysates were extracted 2 hr after MES treatment, and analyzed using the indicated antibodies. (C) Differentiated L6, HepG2, Hela and A549 cells and (D) mouse primary skeletal muscle cells were treated with MES (0.1 ms, 1 V/cm, 55 pps) for 10 min. Cell lysates were extracted 2 hr after MES treatment, and analyzed using the indicated antibodies. (E) Differentiated L6 cells were pre-treated with radicicol (10 µM, 1 hr) or STO-609 (10 µM, 1 hr) and then co-treated with MES for 10 min. Cell lysates were extracted 2 hr after MES treatment. Proteins were subjected to Western blotting analysis using the indicated antibodies. (F) Worms were treated once a day with MES for 20 min at larval stage. Worm lysates were extracted 2 hr after MES treatment, and analyzed by Western blotting using the indicated antibodies. β-actin served as internal control. For (A–F), blots shown are representative of 2-3 independent experiments. The number under each blot is the intensity of the blot relative to that of untreated control. N.D., not-detected due to too low intensity.

The *C. elegans* experiments revealed that increased stress resistance and suppression of excess fat accumulation by MES requires LKB1-AMPK pathway. But it remains unclear whether MES, like other biological or mechanical stresses, activates AMPK. To address this point, we treated mammalian cell lines and *C. elegans* with MES. Interestingly, MES increased the phosphorylation of AMPKα in rat skeletal muscle cell L6 (Fig. 5A). Intriguingly, MES at 0.1-ms pulse width, but not other pulse widths, clearly induced AMPKα phosphorylation (Fig. 5B), similar to the condition used in our previous works [6], [13]. MES-induced phosphorylation of AMPKα was also observed in human hepatocyte HepG2 cell and in mouse primary skeletal muscle cell (Fig. 5C–D), but not in cell lines lacking LKB1 such as human cervical carcinoma HeLa cells and lung carcinoma A549 cells (Fig. 5C) [28], [29]. Pre-treatment with inhibitor of another AMPK-kinase CaMKKβ, STO-609, did not abolish the MES-induced phosphorylation of AMPKα, but pre-treatment with LKB1 inhibitor, radicicol, attenuated the MES-induced AMPKα phosphorylation (Fig. 5E). Therefore, MES-induced AMPK activation was dependent on LKB1, not on CaMKKβ. Furthermore, MES increased the phosphorylation of AMPKα homolog AAK-2 in N2 worms but not in *aak-2* mutant and *par-4* mutant worms (Fig. 5F). These results showed that MES at 0.1-ms pulse width induced AMPK activation both in mammalian cells and *C. elegans*.

### MES slightly decreases mitochondrial membrane potential and intracellular ATP level

**Figure 6:**
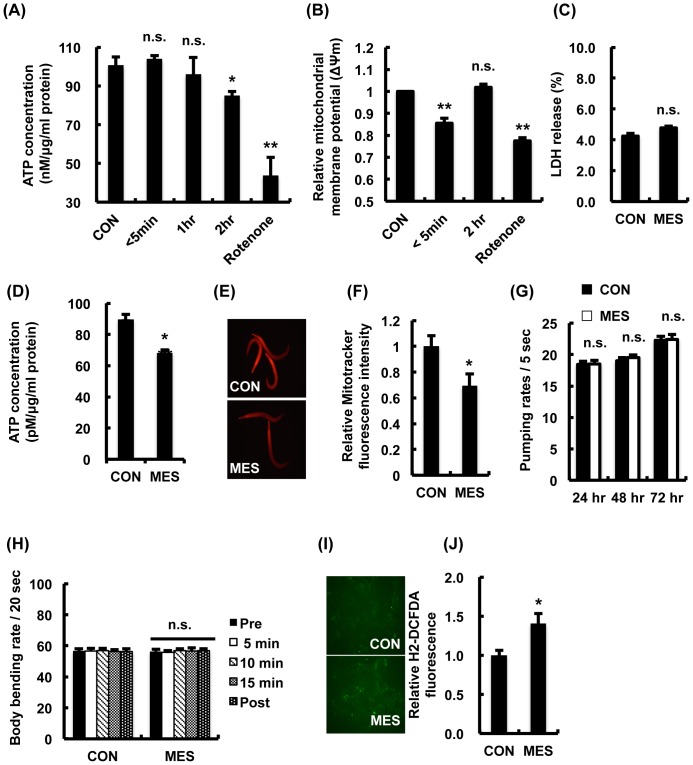
MES transiently decreases mitochondrial membrane potential. (A) Differentiated L6 cells were treated with MES (0.1 ms, 1 V/cm, 55 pps) for 10 min. Then cells were incubated for the indicated time and stained with mitochondrial membrane potential sensor JC-1. Stained cells were subjected to flow cytometry. Rotenone was used as negative control. Data are presented as mean ± SE. ^**^P<0.01 versus control, n.s., not significant, assessed by one-way ANOVA. (B) Differentiated L6 cells were treated with MES for 10 min. Then cells were incubated for the indicated time and subjected to ATP assay. Rotenone was used as negative control. Data are presented as mean ± SE. ^*^P<0.05, ^**^P<0.01 versus control, n.s., not significant, assessed by one-way ANOVA. (C) Differentiated L6 cells were treated with MES for 10 min. LDH release was assessed 24 hr after MES treatment. Data are presented as mean ± SE. n.s., not significant. (D) Wild type N2 worms were treated once a day with MES for 20 min at larval stage. After the last treatment, worm lysates were extracted and subjected to ATP assay. Data are presented as mean ± SE. ^**^P<0.01 versus control. (E) Wild type N2 worms were treated once a day with MES for 20 min at larval stage. After the last treatment, worms were stained with Mitotracker Red CMXRos and observed under a fluorescent microscope. (F) Relative fluorescent intensity of Mitotracker Red CMXRos in (E) was quantified. Data are presented as mean ± SE. ^*^P<0.05 versus control, assessed by unpaired t-test. (G) Wild type N2 worms were treated once a day with MES (0.1 ms, 2 V/cm, 55 pps) for 20 min at larval stage. After each treatment, we recorded and counted the number of pharyngeal pumping activity per 5 seconds for individual worms placed in NGM plates. Data are presented as mean ± SE. n.s., not significant. (H) Wild type N2 worms were treated once a day with MES for 20 min at larval stage. After the last treatment, we recorded at 0, 5, 10, 15, 20 min and counted the number of body bends per 20 seconds for individual worms placed in M9 buffer. Data are presented as mean ± SE. n.s., not significant. (I) Differentiated L6 cells were treated with MES (0.1 ms, 1 V/cm, 55 pps) for 10 min and then cells were stained with ROS indicator H_2_-DCFDA and observed under a fluorescent microscope. (J) Relative fluorescent intensity of H_2_-DCFDA in (I) was quantified. Data are presented as mean ± SE. ^*^P<0.05 versus control, assessed by unpaired t-test. For (A-J), data shown are representative of two independent experiments.

Because increasing the intracellular AMP: ATP ratio promotes phosphorylation by the upstream kinase LKB1 [30], we next investigated the intracellular ATP content following MES treatment. In L6 cells, MES slightly reduced intracellular ATP level by approximately 15% of the untreated control cells at 2 hr after MES treatment (Fig. 6A). We also investigated whether MES decreased mitochondrial membrane potential because decline of mitochondrial membrane potential is expected to lower cellular ATP level [31]. We measured in L6 cells the fluorescence ratio of the JC-1 dye, a lipophilic fluorophore that forms J-aggregates in proportion to the intra-mitochondrial concentration of JC-1 [32]. The results showed that MES slightly and transiently decreased the mitochondrial membrane potential (Fig. 6B). Severe decrease of the mitochondrial membrane potential causes apoptotic cell death [33], but MES did not have cytotoxic activity in L6 cells (Fig. 6C). Moreover, ATP measurement analysis showed that MES also slightly reduced the intracellular ATP level in N2 worms (Fig. 6D). We stained the worms with MitoTracker Red, a specific measurement dye for mitochondrial membrane potential. The fluorescence intensity was slightly decreased in MES-treated N2 worms (Fig. 6E-F). Because caloric restriction and increased locomotive activities are factors that affect intracellular ATP level [34], we measured pharyngeal pumping rate and body bending rate in MES-treated N2 worms. We found no difference in these phenotypes in MES-treated N2 worms compared with control worms (Fig. 6G–H). Another possibility that causes the decrease of intracellular ATP level by MES is reactive oxygen species (ROS) production, which is associated with a drop in mitochondrial membrane potential [35]. We determined intracellular ROS generation after MES treatment using H_2_-DCFDA, a detector of ROS. The result showed that MES increased the level of ROS (Fig. 6I-J). These data suggest that MES slightly decreased the mitochondrial membrane potential possibly via ROS generation without inducing cytotoxicity, subsequently decreasing the intracellular ATP concentration, followed by the activation of LKB1-AMPK signaling pathway.

## Discussion

Exogenous electrical stimulation has been applied as treatment modality in clinical setting for a variety of diseases with few side effects [1]. But studies dissecting the molecular mechanisms involved in its effects are sparse. In this study, we firstly and genetically analyzed the association between the effects of electrical current and its molecular mechanism by utilizing genetic animal model *C. elegans*. Application of imperceptible voltage (1∼2 V/cm) and short-pulse width (0.1 ms) MES increased the stress resistance in wild-type *C. elegans* (Fig. 1A–B). In many organisms, mild exposure or pre-conditioning to a stressor increases resistance to subsequently high intensity of the same or different stressors [36], an adaptive response termed hormesis. In *C. elegans*, pretreatment with non-lethal stress such as mild UV irradiation, heat shock, hypoxia, oxidative stress and caloric restriction can promote stress resistance and metabolic health [37]–[39]. The molecular mechanisms underlying hormesis are not completely understood, but are thought to involve stress responses that activate anti-stress systems or metabolic responses [40]. Thus, MES may be a novel approach of inducing hormetic response. In fact, our previous studies have shown that pre-treatment with MES in combination with heat shock protected the liver from hepatic ischemia/reperfusion injury and the stomach from indomethacin-induced gastric ulcer in mice [23], [41]. In the present study, we performed all experiments on the larval- to young adult-stage *C. elegans* since previous reports indicated that hormetic response exists in young but not in mature or old *C. elegans* [42]. One of the observed beneficial effects of MES in *C. elegans* is the extension of median life span. Increased median life span can be an important indicator that a specific disease has been prevented. Taken together, our data suggest that MES increases the median life span by increasing stress resistance and preventing stress-induced damage.

The MES-induced stress resistance and suppression of excess fat accumulation are due to the regulation of the transcription factors DAF-16, SKN-1 and SBP-1 (Fig. 1C–G, 2D–F), consequently altering their downstream genes' expression (Fig. 1I–N, 2C). The altered activity of these transcription factors by MES is likely mediated through the activation of AMPK signaling pathway because these transcription factors are regulated by AAK-2/AMPK in mammal and *C. elegans* [43]. SKN-1/Nrf2 was activated by metformin to induce oxidative stress response and dietary restriction-like state to extend health span [20]. Increased activity of DAF-16 induced by AAK-2 modulated the central pathway in the regulation of stress resistance and life span by dietary restriction [39]. Additionally, nuclear translocation and transcriptional activity of SREBP was inhibited by activated AMPK [44]. Activation of AMPK by LKB1 requires an increase in AMP: ATP ratio because binding of AMP to AMPK alters the conformation of heterotrimeric AMPK, making it a better substrate for LKB1 [30]. Intriguingly, the intracellular ATP level was down-regulated by MES, and MES slightly decreased the mitochondrial activity (Fig. 6A–F). The effects of MES were not observed in AMPK homolog *aak-2* mutant worms (Fig. 3), and its upstream kinase LKB1 homolog *par-4* mutant worms (Fig. 4), suggesting that LKB1-AMPK signaling pathway is involved in MES-induced stress resistance. However, it is also possible that due to the inherent short life span and hypersensitivity of these mutant worms to stress, the effects of MES could not be clearly detected.

A number of intracellular modulators such as ROS, fatty acid, proton, Na^+^, K^+^ and Ca^2+^ have been reported as factors that can suppress mitochondrial membrane potential [45]. Although the precise mechanism how MES inhibits mitochondrial membrane potential is still unclear, we found that MES increased the level of ROS (Fig. 6I–J). Our results suggested that MES activates LKB1-AMPK signaling pathway via ROS generation, slight inhibition of mitochondrial membrane potential and decreased ATP production. AMPK can also be activated by CaMKKβ, which is triggered by a rise in the intracellular Ca^2+^ concentration [25]. However, our previous study showed that MES had no effect on membrane depolarization and intracellular Ca^2+^ influx [8]. In addition, pre-treatment with CaMKKβ inhibitor STO-609 did not inhibit MES-induced AMPK activation (Fig. 5C). Collectively, these results suggested that MES activates AMPK through LKB1, not through CaMKKβ. Because MES or low-intensity electrical current impacts on many cellular processes and functions, we cannot rule out the possibility that signaling pathways other than LKB1-AMPK are affected by MES as well. Furthermore, the receptor for MES at 0.1-ms pulse width is unknown. Our preliminary investigations suggested the possibility that some members of the transient potential receptor (TRP) family might be involved in the effects of MES (data not shown). Deeper explorations might shed light on the role of TRP channels in the recognition and transduction of MES.

Drugs such as biguanide and thiazolidinedione, which activate AMPK, are clinically applied for type 2 diabetes mellitus and obesity because of the critical action of AMPK on glucose and lipid metabolism. Furthermore, recent studies indicate that AMPK is an attractive therapeutic target for the treatment of AMPK dysfunction-related diseases like Alzheimer's disease, kidney dysfunction and inflammation [17], [46]. Based on our previous [6], [10], [11] and current studies, MES might be a promising therapeutic approach for various diseases, at least in part, through activation of LKB1-AMPK signaling pathway.
